# Right Heart Pressure Increases after Acute Increases in Ambient Particulate Concentration

**DOI:** 10.1289/ehp.11230

**Published:** 2008-05-28

**Authors:** David Q. Rich, Ronald S. Freudenberger, Pamela Ohman-Strickland, Yong Cho, Howard M. Kipen

**Affiliations:** 1 School of Public Health, University of Medicine and Dentistry of New Jersey, Piscataway, New Jersey, USA; 2 Environmental and Occupational Health Sciences Institute, University of Medicine and Dentistry of New Jersey–Robert Wood Johnson Medical School and Rutgers University, Piscataway, New Jersey, USA; 3 University of Medicine and Dentistry of New Jersey–Robert Wood Johnson Medical School, New Brunswick/Piscataway, New Jersey, USA; 4 Center for Advanced Heart Failure, Lehigh Valley Hospital and Health Network, Allentown, Pennsylvania, USA; 5 Medtronic Inc., Minneapolis, Minnesota, USA

**Keywords:** air pollution, diastolic, heart, pressure, pulmonary artery, right ventricle

## Abstract

**Objectives:**

We explored the association between acute changes in daily mean pulmonary artery (PA) and right ventricular (RV) pressures and concentrations of ambient fine particulate matter [PM with aerodynamic diameter ≤2.5 μm (PM_2.5_)] as an explanation for previous associations between congestive heart failure (HF) hospital admissions and PM.

**Materials and methods:**

In the Chronicle Offers Management to Patients with Advanced Signs and Symptoms of Heart Failure (COMPASS-HF) trial, to see whether management of ambulatory HF could be improved by providing continuous right heart pressure monitoring to physicians, the Chronicle Implantable Hemodynamic Monitor (Medtronic, Inc., Minneapolis, MN, USA) continuously measured multiple right heart hemodynamic parameters, heart rate, and activity trends in subjects with moderate/severe HF. Using these trial data, we calculated daily mean pressures, using only those time intervals where the subject was not physically active (*n* = 5,807 person-days; *n* = 11 subjects). We then studied the association between mean daily PA/RV pressures and mean ambient PM_2.5_ concentrations on the same day and previous 6 days.

**Results:**

Each 11.62-μg/m^3^ increase in same-day mean PM_2.5_ concentration was associated with small but significant increases in estimated PA diastolic pressure [0.19 mmHg; 95% confidence interval (CI), 0.05–0.33] and RV diastolic pressure (0.23 mmHg; 95% CI, 0.11–0.34). Although we saw considerable differences in the magnitude of response by COMPASS-HF randomization group (total data access for physicians vs. blocked clinician access), season, left ventricular ejection fraction, and obesity, these effects were not significantly different.

**Conclusions:**

These pilot study findings provide a potential mechanism for previous findings of increased risk of HF associated with ambient PM. However, because of the small number of subjects, a larger study is needed for confirmation.

Increased incidence of specific acute cardiovascular outcomes, including myocardial infarction, ventricular arrhythmia, episodes of atrial fibrillation, ischemic stroke, and heart failure (HF), have been reported to occur immediately after as little as 1–2 hr of increased particulate matter (PM) concentration (Peters et al. 2001; Rich et al. 2006b), as well as 1–2 days later (Burnett et al. 1999; D’Ippoliti et al. 2003; Morris and Naumova 1998; Morris et al. 1995; Peters et al. 2000, 2005; Rich et al. 2005, 2006a; Schwartz and Morris 1995; Symons et al. 2006; Wellenius et al. 2005a, 2005b, 2006; Wong et al. 1999; Zanobetti and Schwartz 2005). However, not all studies have reported these acute to subacute responses (Levy et al. 2001; Rich et al. 2004; Sullivan et al. 2005; Vedal et al. 2004). Recent epidemiologic, animal, and controlled human exposure studies have suggested that PM air pollution may elicit changes in subclinical indices, such as autonomic function, inflammation, endothelial dysfunction, and thrombotic tendency within hours after air pollution exposures, which may contribute to the increased incidence of cardiovascular disease observed (Brook et al. 2002; Gold et al. 2000; Liao et al. 1999; Magari et al. 2001, 2002; Mills et al. 2005, 2007; Nemmar et al. 2002, 2003; Riediker et al. 2004; Ruckerl et al. 2006; Tornqvist et al. 2007; Tunnicliffe et al. 2001; Wellenius et al. 2003).

Wellenius et al. (2005a) reported small but statistically significant increases in the risk of HF hospital admissions associated with increases in same-day PM_10_ and nitrogen dioxide concentration in Pittsburgh, Pennsylvania. In a later multicity study, these same investigators reported a 0.72% [95% confidence interval (CI), 0.35–1.10%] increase in risk of HF admission for each 10-μg/m^3^ increase in same-day ambient PM _10_ concentration (Wellenius et al. 2006). These Medicare database admission studies demonstrated acute cardiovascular responses to air pollution that require an exploration of possible physiologic correlates both for verification and for clues as to mechanism. We hypothesized that hospital admissions for decompensation of HF that were triggered by air pollution would be associated with more frequent subclinical increases in pulmonary arterial (PA) diastolic and right ventricular (RV) pressures. Such pressure increases could ultimately lead to emergency department/hospital admission when individuals are vulnerable due to the presence of other established HF exacerbation triggers (e.g., volume overload, electrolyte status, decreased oxygenation, rhythm disturbance, exertion, decreased myocardial contractility, suboptimal medical therapy). If PM has a causal role in HF admissions because of acute pressure increases, then direct passive measurement of these specific pressures should be a much more sensitive and upstream measure of stress on the cardiopulmonary system than hospital admissions for HF, which presumably depend on a confluence of exacerbating factors.

Therefore, we coupled passively monitored data of continuous PA diastolic and RV pressure data in HF patients implanted with the Chronicle Implantable Hemodynamic Monitor (Medtronic, Inc., Minneapolis, MN) and ambient fine PM [aerodynamic diameter ≤ 2.5 μm (PM_2.5_)] measurements made at monitoring stations throughout New Jersey. In this pilot study to investigate the feasibility of such an approach to examine mechanisms underlying previously reported associations between air pollution and HF, we examined the association between daily mean PM_2.5_ concentrations and daily mean PA and RV pressures.

## Materials and Methods

### Study population and outcomes

We studied class III HF patients who were participating in the COMPASS-HF (Chronicle Offers Management to Patients with Advanced Signs and Symptoms of Heart Failure) trial and were implanted with the Chronicle at either Robert Wood Johnson University Hospital in New Brunswick, New Jersey, or Beth Israel Medical Center in Newark, New Jersey (*n* = 13 subjects). In this trial, the Chronicle continuously measured and stored intracardiac pressure, body temperature, physical activity, and heart rate. This device has been described in detail previously (Bourge et al. 2008). Briefly, the device includes a programmable unit similar to the pulse generator of a pacemaker, and a transvenous lead with a sensor near its tip to measure intracardiac pressures. The device is positioned subcutaneously in the pectoral area. The lead is placed transvenously with its tip located in the RV outflow tract or high septum. Approximately every 8 min, the device stores the medians of several pressure metrics, including estimated PA diastolic pressure (ePAD), RV diastolic pressure, RV systolic pressure, mean PA pressure (MPAP), physical activity, and heart rate (beats per minute). ePAD is defined as the RV pressure at the time of pulmonary valve opening and is correlated (*r* = 0.84) with actual PA pressure (Magalski et al. 2002; Ohlsson et al. 1995; Zile and Brutsaert 2002). Physical activity level was measured by an accelerometer in the chest wall device. Any physical activity sensed by the accelerometer is measured and averaged over the programmed storage interval (i.e., 8 min). A physical activity of 0 means that the sensor did not detect any activity during that interval.

Each week, these measurements were transmitted by patients via telephone line and subsequently downloaded to each patient’s cardiologist using the Internet. As part of the COMPASS-HF trial, subjects were randomized subjects to either “total clinician access,” which meant the cardiologist could view these data weekly and, if necessary, prescribe changes in medications or therapy, or “blocked clinician access,” which meant the cardiologist could not view these pressure data and could monitor the subject only during regularly scheduled clinic visits (i.e., the normal standard of care). Details of this trial have been published previously (Bourge et al. 2008). A barometric pressure monitor (Chronicle Tracker, Medtronic, Inc.) worn by the patient also made continuous measurements and stored values for the same time intervals as the heart pressure readings. As part of the COMPASS-HF trial, ejection fraction was obtained by standard clinical echocardiography. Heart failure etiology was based on clinical criteria. If there was known coronary artery disease or history of myocardial infarction, patients were categorized as ischemic. Patients without these conditions were categorized as non ischemic.

Of the original 13 study subjects implanted with the Chronicle, we excluded one patient who lived in New York because we could not match a New Jersey air pollution monitor to that person. We also excluded a second subject who died of septic shock 24 days after device implantation, leaving 11 subjects for analysis. For each subject, we included all pressure data from the date of implant through the date of death or last data transmittal before 11 January 2007 [range, 358–1,090 days; median, 846 days (2.32 years)]. For our primary analysis examining changes in daily pressure and daily PM_2.5_ concentration, we excluded all intervals where the subject’s physical activity was > 0 and then calculated mean PA and RV pressures for each day of observation, leaving *n* = 5,807 person-days (*n* = 11 subjects) for analysis. The University of Medicine and Dentistry of New Jersey Institutional Review Board (New Brunswick and Piscataway campuses) approved this study.

### PM and weather measurements

PM_2.5_ was measured continuously in New Brunswick, Camden, Elizabeth, Jersey City, and Rahway, New Jersey, during the study period by the New Jersey Department of Environmental Protection (Trenton, NJ) using a tapered element oscillating microbalance. We retrieved hourly concentrations at these stations from the U.S. Environmental Protection Agency (EPA) website (U.S. EPA 2007). For each subject, we calculated the distance between each PM_2.5_ monitor and the subject’s residence, assigning PM_2.5_ measurements from the closest monitor to their residence. We assigned all subjects PM_2.5_ measurements from either the New Brunswick or Elizabeth monitoring sites (median distance, 13.4 km; range, 7.0–34.2 km).

Temperature and dew point were measured hourly at the Newark, Caldwell, Somerset, and Trenton airports during the study period. We used the airport monitor closest to each subject’s residence to provide the weather observations for that subject during the study period. For each day, we calculated apparent temperature (Kalkstein and Valimont 1986; O’Neill et al. 2007; Steadman 1979; Zanobetti and Schwartz 2005) as a measure of each subject’s perceived air temperature given the humidity [apparent temperature = –2.653 + (0.994 × air temperature) + (0.0153 × dewpoint2)] and used these values in all analyses.

### Statistical analysis

We used a two-step modeling process to estimate the change in daily mean ePAD associated with each incremental increase in same-day (i.e., lag 0) mean PM_2.5_ concentration. In the first step, each subject appeared to have different long-term patterns in ePAD during the follow-up period (data not shown). Therefore, we removed long-term trends in ePAD separately for each person via spline-based nonparametric regression. We regressed a natural cubic spline (3 degrees of freedom) of time against ePAD and obtained residuals from this model to be used as the outcome in the second step of the analysis. We found that higher degrees of freedom were not necessary for modeling the long-term trends and did not substantially alter the results in the second step of the analysis (data not shown). In the second step, focusing on average short-term pressure changes associated with same-day PM_2.5_ concentration, we used a repeated-measures model with these residuals as the response, combining all subjects’ data into a single model. We regressed fixed factors of same-day PM_2.5_ concentration, day of week, and month, as well as quadratic effects of apparent temperature and barometric pressure, against these residuals, using a first-order autocorrelation structure, such that we modeled measurements separated by *d* days to have a correlation of ρ*_d_*, where ρ represents the correlation between measurements from 2 successive days. We then repeated this procedure to examine separately the changes in daily RV diastolic pressure, RV systolic pressure, and MPAP associated with each 11.62 μg/m^3^ increase (the interquartile range observed during the study period) in same-day mean PM_2.5_ concentration. Relative to compound symmetry, we found the first-order autocorrelation structure provided a better fit for the data as determined by Akaike’s information criterion.

Next, to evaluate whether the assumption of a linear exposure response function was justified, we examined the concentration–response using quintiles of PM_2.5_ and performed a test of trend. Last, to evaluate whether any association between daily right heart pressure and same-day PM_2.5_ concentration was independent of previous days’ mean PM_2.5_ concentrations, we repeated this two-stage modeling process including the mean PM_2.5_ concentrations from lag days 0–6, rather than just lag 0, in the same model. For each analysis, we present the unit change in right heart pressure associated with each 11.62 μg/m^3^ increase in PM_2.5_ concentration, and its 95% CI.

We also evaluated effect modification of any pressure–PM_2.5_ association by randomization group (total clinician access vs. blocked clinician access), left ventricular ejection fraction (LVEF) at implantation (≥ 45% vs. < 45%), season (winter vs. summer), and body mass index (BMI) at implantation (obese, BMI ≥ 30, vs. nonobese, BMI < 30), by adding an interaction term (effect modifier ×PM_2.5_) to the mixed model described above. We present the unit change in right heart pressure associated with each 11.62 μg/m^3^ increase in PM_2.5_ concentration for each category of each potential effect modifier. We conducted all analyses using SAS/STAT software, version 9.1.3 for Windows (SAS Institute Inc., Cary, NC).

## Results

Subjects were predominantly white (73%), and obese (73% had BMI ≥ 30) and ranged in age from 25 to 68 years (median, 57 years). All but two subjects had LVEFs < 45% at Chronicle implantation, and seven (64%) also had an implantable cardioverter defibrillator (ICD) or biventricular ICD (Bi-V ICD) (Table 1). All subjects (*n* = 11) were taking beta-blockers and diuretics during the study period, with lower but substantial proportions taking angiotensin converting enzyme inhibitors (*n* = 9), amiodarone (*n* = 4), digoxin (*n* = 8), anticoagulants (*n* = 6 ), aspirin (*n* = 6), and antihyperlipidemia medications (*n* = 4).

We found significant increases in daily mean ePAD and RV diastolic pressure, and nonsignificant increases in daily mean RV systolic pressure and MPAP, associated with each 11.62-μg/m^3^ increase in same-day mean PM_2.5_ concentration, after adjusting for long-term time trends, calendar month, weekday, and apparent temperature (Table 2). Because we found larger, significant associations with ePAD and RV diastolic pressure, we restricted all further analyses to these pressure measurements.

**Figure 1:**
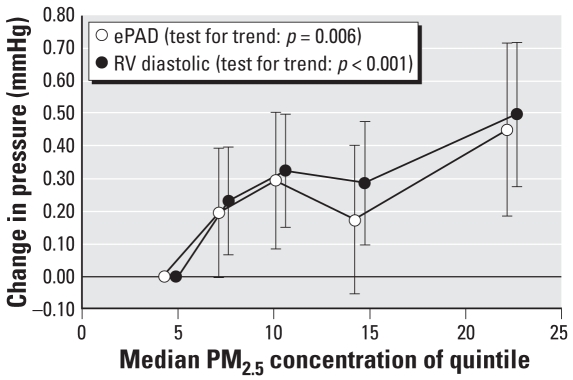
Change in mean daily ePAD (mmHg) and RV diastolic pressure (mmHg) per quintile of mean daily PM_2.5_ concentration on lag day 0 (*n* = 11 subjects). Error bars are 95% CIs. Tests for trend were statistically significant: RV diastolic pressure, *p* < 0.001; ePAD, *p* = 0.006.

**Figure 2:**
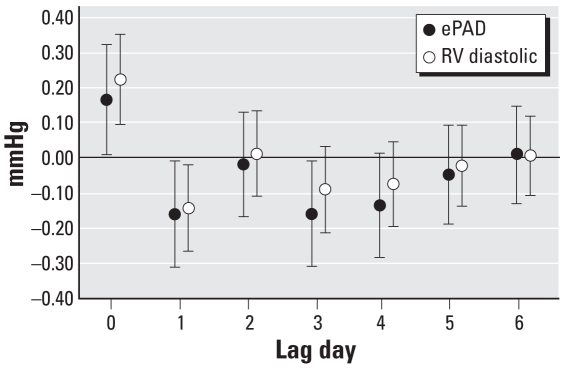
Change in mean daily ePAD (mmHg) and RV diastolic pressure (mmHg) per 11.62-μg/m^3^ increase in mean daily PM_2.5_ concentration on lag days 0–6 (*n* = 11 subjects). Error bars are 95% CIs

Changes in RV diastolic pressure and ePAD generally increased with quintiles of PM_2.5_ (Figure 1), and the tests for trend were statistically significant for both pressures (RV diastolic pressure, *p* < 0.001; ePAD, *p* = 0.006). When we included the mean PM_2.5_ concentrations for lag days 1–6 in the same model, ePAD and RV diastolic pressure increases associated with each 11.62-μg/m^3^ increase in same-day (lag 0) mean PM_2.5_ concentration were not substantially attenuated and remained statistically significant (ePAD: 0.17 mmHg; 95% CI, 0.02–0.32; RV diastolic pressure: 0.23 mmHg; 95% CI, 0.10–0.35) (Figure 2).

We found no statistically significant effect modification of the PM_2.5_–RV diastolic pressure association by access randomization group, LVEF at implantation, season, or BMI at implantation (Table 3). However, the unit change in RV diastolic pressure was largest for the blocked clinician access randomization group compared with the total clinician access group, subjects with LVEF ≥ 45% at implantation compared with subjects with LVEF < 45%, winter versus summer person-days, and obese subjects compared with nonobese subjects (Table 3).

## Discussion

In a pilot study using continuous PA and RV pressures in 11 class III HF patients and ambient measurements of PM_2.5_ at nearby monitoring stations, we observed small, but statistically significant increases in mean daily ePAD and RV diastolic pressure (0.19 and 0.23 mmHg, respectively) associated with each 11.62-μg/m^3^ increase in mean PM_2.5_ concentration on the same day. These increases were not attenuated when controlling for the previous 6 lag days of mean PM_2.5_ concentration. Further, the concentration–response functions were generally linear. However, we found no statistically significant effect modification by access randomization group, LVEF at implantation, obesity, or season. These findings add mechanistic plausibility to previous studies’ reports of increased hospital admissions for HF associated with ambient PM concentrations in the previous few days (Burnett et al. 1999; Morris et al. 1995; Schwartz and Morris 1995; Symons et al. 2006; Wellenius et al. 2005a, 2006).

The observed pattern of lag day response (strongest effect on lag day 0, a negative effect on lag day 1–3, and little change in pressure for lag days 4–6) is a pattern similar to that observed by Wellenius et al. (2006). In their study of Medicare hospital admissions for HF, they reported a 0.72% increase in the risk of hospital admission for HF associated with each 10-μg/m^3^ increase in PM_10_ on lag day 0, a negative association on lag day 1, and no association on lag days 2 and 3.

Ambient PM may trigger pathophysiologic mechanisms by several means, which ultimately lead to acute exacerbation of HF. These include direct depressant effects on the myocardium secondary to ischemia (Pekkanen et al. 2002; Wellenius et al. 2003), arrhythmia (Rich et al. 2005, 2006a), PA vasoconstriction (observed in rodent models; Batalha et al. 2002), and reduced alveolar fluid clearance (Mutlu et al. 2006). These nonischemic mechanisms may be of particular importance because most of our subjects did not have an underlying ischemic mechanism for their HF (*n* = 7). The ultimate effect of any of those pathways would be an increase in right heart pressures, leading to hospitalization if it is amplified and prolonged without compensation or treatment. The pressure changes resulting in a hospitalization for HF are certainly much greater than those observed on average for these 11 subjects, perhaps between one and two orders of magnitude. One possible explanation for the muted pressure response to ambient PM_2.5_ in these 11 subjects is better management, with state-of-the-art medical therapy (a requirement of the COMPASS-HF trial), than that received by the population of Medicare patients examined by Wellenius et al. (2006). Thus, our lack of dramatic pressure changes does not negate the likelihood that the small changes we observed could trigger larger changes in an HF patient more particularly prone to decompensation. Ultimately, the small changes we observed and the limited number of subjects in our analysis suggest the need for further study in larger panels/cohorts to validate RV pressure increases as a mechanism for acute air pollution effects on HF exacerbations.

Although our study had strengths, including passive continuous monitoring of PA and RV pressures in moderate to severe HF patients, it also had several limitations. First, exposure mis-classification was likely because we used ambient measurements of PM_2.5_ to approximate each subject’s exposure, rather than personal measurements. However, because this exposure error is likely nondifferential with respect to heart pressure measurements, our reported effect estimates are likely underestimates.

Second, it is possible that residual confounding by meteorology conditions or an unmeasured confounder may be responsible for the effects observed. However, that factor would have to both be correlated with daily mean PM_2.5_ concentrations measured at the monitoring stations and increases in daily mean PA and RV pressures in these 11 subjects. Thus, it seems unlikely that other meteorologic conditions not correlated with those included in our analysis (temperature, relative humidity, and barometric pressure) or other acutely time-varying, non-weather-related factors could explain the results observed.

Third, although we retained 5,807 person-days in our analysis, these were among only 11 subjects, resulting in reduced statistical power in our main analyses and even less power in our assessment of effect modification by subject specific characteristics (e.g., access randomization group, obesity, and LVEF at implantation) and season. However, although we found no statistically significant effect modification by these characteristics, there were considerable differences in the magnitude of response between each level of these characteristics (e.g., total clinician access vs. blocked clinician access, obese vs. nonobese, LVEF ≥ 45% vs. LVEF < 45%, winter vs. summer), suggesting that with a larger sample size these differences may have been statistically significant. The previously reported associations between obesity and susceptibility to air pollution (Chen et al. 2007; Zeka et al. 2006) as well as increased PM-related health effects in winter time (Becker et al. 2005) are consistent with our findings.

In this pilot study of the acute effects of ambient PM_2.5_ concentrations on a likely precursor of hospital admissions for HF exacerbation (PA and RV pressure increases), we demonstrated the feasibility of linking ambient air pollution data to continuously measured right heart pressures in HF patients. Further, we found significant increases in daily ePAD and RV diastolic pressure associated with increases in same-day ambient PM pollution concentrations. We have planned confirmation in a larger sample with more varied geography and therefore likely different sources and composition of PM.

## Figures and Tables

**Table 1:** Characteristics of study population (*n* = 11).

ID no.	Sex	Race	Height (m)	Weight (kg)	BMI (kg/m^2^)	Age at implant (years)	HF etiology	Heart rate at implant (bpm)	LVEF at implant	Other implanted device
01	Female	White	1.60	78.9	30.8	60	Ischemic	76	65	None
02	Female	White	1.68	67.1	23.9	57	Nonischemic	71	20	None
03	Female	White	1.60	97.5	38.1	33	Nonischemic	82	15	ICD
04	Female	Hispanic	1.65	92.1	33.8	42	Nonischemic	84	40	None
05	Female	Black	1.55	74.8	31.2	49	Nonischemic	48	45	None
06	Male	White	1.83	88.4	26.4	65	Nonischemic	76	20	Bi-V ICD
07	Male	White	1.70	92.1	31.8	67	Ischemic	80	40	ICD
08	Male	White	1.75	117.9	38.4	61	Nonischemic	72	20	Bi-V ICD
09	Female	Black	1.65	130.6	47.9	25	Nonischemic	94	15	ICD
10	Male	White	1.73	73.9	24.8	53	Ischemic	84	35	ICD
11	Male	White	1.75	100.2	32.6	68	Ischemic	56	22	ICD

**Table 2:** Change in mean daily pressure (mmHg) per 11.62-μg/m^3^ increase in mean PM_2.5_ concentration on the same day (*n* = 11 subjects).

Outcome	No. of person-days	Change in pressure (mmHg)	95% CI	*p*-Value
ePAD	5,807	0.19	0.05 to 0.33	0.01
RV diastolic pressure	5,807	0.23	0.11 to 0.34	< 0.001
RV systolic pressure	5,807	0.12	−0.07 to 0.31	0.23
MPAP	5,667	0.12	−0.05 to 0.28	0.16

**Table 3:** Change in mean daily RV diastolic pressure (mmHg) per 11.62-μg/m^3^ increase in mean PM_2.5_ concentration on the same day, by level of effect modifier (*n* = 11 subjects; *n* = 5,807 person-days).

Effect modifier	No. of person-days	Change in pressure (mmHg)	95% CI	Interaction term *p*-value
LVEF (%)				
≥ 45	1,006	0.28	0.05 to 0.51	0.61
< 45	4,801	0.22	0.10 to 0.34	
Obesity				
Obese (BMI ≥ 30)	3,838	0.27	0.14 to 0.40	0.22
Nonobese (BMI < 30)	1,969	0.15	−0.02 to 0.32	
Access randomization group				
Total clinician access	2,305	0.16	0.00 to 0.32	0.22
Blocked clinician access	3,502	0.28	0.14 to 0.41	
Season^a^				
Winter	1,297	0.31	0.09 to 0.54	0.33
Summer	1,374	0.15	−0.09 to 0.39	

a Winter, December–February. Summer, June–August. We excluded person-days occurring in the spring or autumn from this analysis.
